# Calcium intake and the associations with faecal fat and energy excretion, and lipid profile in a free-living population

**DOI:** 10.1017/jns.2017.55

**Published:** 2017-09-19

**Authors:** Louise Kjølbæk, Janne K. Lorenzen, Lesli H. Larsen, Arne Astrup

**Affiliations:** Department of Nutrition, Exercise and Sports, Faculty of Science, University of Copenhagen, Rolighedsvej 26, 1958 Frederiksberg C, Denmark

**Keywords:** Dietary calcium, Faecal fat excretion, Faecal energy excretion, Lipid profile, Cholesterol, BP, blood pressure, CHOL, cholesterol, Q1, first quartile, Q3, third quartile, RCT, randomised controlled trial

## Abstract

The aim of the present study was to investigate the associations between the habitual Ca intake and faecal fat and energy excretion as well as blood lipid profile in free-living normal-weight and overweight individuals. The participants were enrolled for an 8-d period where data from a 7-d diet registration (days 1–7), a 5-d faeces collection (days 3–7), a 2-d urine collection (days 5–7), and anthropometric measurements and a fasting blood sample (day 8) were collected. Analyses showed that dietary Ca intake (g/10 MJ per d) was positively associated with excretion of faecal fat (*P* = 0·004) and energy (*P* = 0·031) when adjusted for BMI, age, sex and intake of Ca-containing supplements. However, after adjustment for intake of fibre, the effect of Ca intake disappeared. Nevertheless, total cholesterol (CHOL) and LDL-CHOL concentrations were associated negatively with Ca intake (*β* −0·62 (95 % CI −0·96, −0·28) mmol/l, *P* < 0·001, and *β* −0·49 (95 % CI −0·78, −0·20) mmol/l, *P* = 0·001, respectively, per 1000 mg/10 MJ per d increase in Ca intake). In conclusion, incorporation of Ca-rich food products in a habitual diet was associated with reduced total CHOL and LDL-CHOL concentrations, which may lower the risk of CVD in the long term.

More than 1·9 billion adults are overweight or obese worldwide^(^^1^^)^ and obesity is considered a global health issue because it increases the risk of CVD and diabetes^(^^1^^)^. Weight-loss (maintenance) diets have been intensively investigated, especially with focus on the energy-providing macronutrients^(^^2^^–^^5^^)^. However, the micronutrient Ca has been associated with reduced BMI since 1984^(^^6^^)^. Subsequently, many studies have found negative associations of dairy product or Ca intakes with anthropometric measurements^(^^6^^–^^23^^)^. Additionally, a higher Ca intake has been associated with reduced insulin concentration^(^^15^^)^, blood pressure (BP)^(^^6^^,^^14^^,^^18^^)^ and reduced risk of the metabolic syndrome^(^^18^^,^^24^^,^^25^^)^ as well as an improved blood lipid profile^(^^7^^,^^18^^)^. Nonetheless, the negative anthropometric association with Ca has not been consistently shown^(^^26^^–^^30^^)^, and the effect of a higher Ca intake on body weight has not been clearly proven by randomised controlled trials (RCT) or meta-analyses^(^^31^^–^^35^^)^.

One of several theories is that Ca binds fat in the intestine, especially the long-chain fatty acids, which causes the formation of insoluble Ca–fatty acid soaps^(^^36^^,^^37^^)^. The decreased solubility of fatty acids and monoacylglycerols in the micelles lowers cholesterol (CHOL) solubility and causes the formation of large aggregates that are unlikely to be absorbed^(^^38^^)^. Thus, a higher Ca intake will result in increased faecal fat excretion^(^^39^^)^. Additionally, Ca precipitates bile acids^(^^40^^)^, which reduces the enterohepatic recycling of bile acids and causes *de novo* synthesis of bile acids from blood CHOL. Overall, this may lead to an improved blood lipid profile.

In observational studies, intakes of dairy products and Ca have been associated with dyslipidaemia^(^^24^^)^, total CHOL^(^^7^^,^^28^^)^, LDL-CHOL^(^^7^^)^, HDL-CHOL^(^^18^^)^, total CHOL:HDL-CHOL ratio^(^^7^^)^ and TAG^(^^24^^)^. However, the findings are not consistent. Some studies have found reduced total CHOL and LDL-CHOL concentrations or increased HDL-CHOL concentration associated with reduced risk of CVD^(^^41^^–^^45^^)^, but others observed that the total CHOL:HDL-CHOL or LDL-CHOL:HDL-CHOL ratio^(^^42^^,^^46^^,^^47^^)^ was a better predictor for CVD than the isolated blood lipid concentrations. In intervention studies, increased Ca intake from fortified products^(^^48^^,^^49^^)^, dietary supplements^(^^17^^,^^50^^–^^53^^)^, milk^(^^34^^,^^54^^,^^55^^)^, cheese^(^^55^^–^^57^^)^, yoghurt^(^^14^^)^ and a mixture of dairy products^(^^15^^,^^17^^,^^35^^,^^50^^,^^58^^–^^60^^)^, as well as Ca intake combined with different fat^(^^61^^)^ or protein^(^^60^^)^ contents, have been investigated. Still, the exact quantity of faecal fat excretion and effect on the lipid profile in relation to the increased Ca intake is inconsistent.

A frequently raised concern is whether the short-term effect of Ca observed in intervention studies will decrease over time due to adaptation to a long-term high habitual Ca intake. Therefore, the aim of the present study was to investigate the association between habitual Ca intake and faecal fat and energy excretion in free-living normal-weight and overweight persons as well as the association between habitual Ca intake and blood lipid profile.

## Participants and methods

### Study design

The study included participants from two trials with similar design for the baseline measurements. In both trials, data were obtained during an 8-d period. The participants’ dietary intake was assessed by a 7-d dietary registration where the participants weighed all foods (days 1–7). Participants recorded physical activity, smoking habits, intake of medication and illness on days 1–7 and women recorded their menstrual cycle. Faecal samples were collected in pre-weighed plastic containers over 5 d (days 3–7) and complete 48-h urine collection was carried out (days 6–7). On day 8, anthropometric measurements and BP measurements were performed and a fasting (12 h) blood sample was drawn at the Department.

Trial I (clinical trial no. NCT01542164; https://clinicaltrials.gov) was an observational study carried out from June 2009 to March 2011. The primary aim was to investigate the associations between habitual Ca intake and faecal fat and energy excretion as well as the association between habitual Ca intake and lipid profile. Individuals were recruited with the goal to obtain forty participants in each of the four groups: (A) BMI > 25 kg/m^2^ and habitual dietary Ca intake ≤800 mg/d; (B) BMI > 25 kg/m^2^ and habitual dietary Ca intake >800 mg/d; (C) BMI ≤ 25 kg/m^2^ and habitual dietary Ca intake ≤800 mg/d; (D) BMI ≤ 25 kg/m^2^ and habitual dietary Ca intake ≤800 mg/d. At inclusion, BMI was calculated from self-reported weight and height and habitual Ca intake was assessed by a self-administered quantitative FFQ highly similar to a FFQ previously validated^(^^62^^)^. Trial II (clinical trial no. NCT01199835; https://clinicaltrials.gov) was an intervention study with a randomised parallel design. Overweight and obese participants (BMI 28–33 kg/m^2^) were randomised to a 24-week intake of a hypoenergetic diet with either a high or a low intake of dairy products. A minimum of forty (and up to fifty) participants were recruited for each group. It was carried out from June 2010 to August 2011 and the aim was to investigate the effect of the hypoenergetic high- *v*. low-dairy diets on weight loss and faecal excretion of fat and energy. Furthermore, the effects on BP, blood lipid profile, lipid oxidation and appetite regulation were investigated (described elsewhere; Bendtsen *et al*.^(^^63^^)^).

Both studies were conducted according to the guidelines laid down in the Declaration of Helsinki and all procedures involving human participants were approved by the responsible regional committee on human experimentation in Denmark (H-B-2009-071 (trial I) and H-3-2010-049 (trial II)). Furthermore, the studies were registered in Clinical Trial and the Danish Data Protection Agency (both studies: 2007-54-0269). Written informed consent was obtained from all participants and both studies were carried out at the Department of Nutrition, Exercise and Sports, Faculty of Science, University of Copenhagen, Denmark.

### Participants

Individuals were recruited through advertisements posted on a university web page (forsøgsperson.dk) and in the local/free newspaper. Potential individuals were pre-screened by telephone in accordance with the inclusion and exclusion criteria and those who fulfilled the criteria were sent written information and the FFQ. Finally, those who fulfilled BMI and habitual Ca criteria were invited to an information meeting where the informed consent was signed.

The inclusion criteria in trial I were 18–50 years and weight stable (±4 kg 4 months prior to study start), and in trial II participants were 18–60 years and had a habitual low Ca intake (<800 mg/d) evaluated by the FFQ. In both studies, individuals were excluded if they were pregnant or lactating, used CHOL-decreasing medication or other types of medication assessed to influence the study outcomes. Furthermore, dieting individuals or individuals with a temporary change in dietary habits as well as individuals with previous and present gastrointestinal diseases were excluded. In trial II individuals with milk allergy, infectious disease and metabolic diseases were excluded too. Smokers and individuals who had taken dietary supplements 6 months prior to (and during the study) were excluded in trial II, whereas in trial I participants recorded their smoking habits and intake of dietary supplements. In trial I, individuals with physical activity more than 8 h/week were excluded. In general, the participants were not allowed to participate in other studies in parallel. However, data collected in trial I constituted the majority of the baseline measurements in trial II; therefore participants were allowed to participate in these two studies at the same time.

As compensation, participants received 1000 Danish Crowns (about US$185) after completion of trial I. The same compensation was provided in trial II, but only if the participant completed a sub-study.

### Outcomes

The primary outcomes were faecal fat and energy excretion. Secondary outcomes were fasting blood lipid profile (total CHOL, LDL-CHOL, HDL-CHOL, NEFA and TAG), resting BP as well as the faecal and urinary excretion of Ca.

### Data collection

In both trials, data were obtained during an 8-d period. In trial I, data were obtained once. In trial II (the RCT), only baseline data were used for analyses.

#### Anthropometry and blood pressure

All participants voided before anthropometric measurements were performed. Body weight (in kg with two decimals) was measured by a digital scale (Lindells). Height was measured twice with 0·5 cm accuracy using a wall-mounted stadiometer (Hultafors) and the average of the two measurements was recorded. BMI was calculated as weight (kg) divided by height squared (m^2^). After 10 min rest, BP was measured by an automatically inflated cuff (Saitama). BP was measured on both arms and the subsequent measurements were performed on the arm with the highest systolic BP. If the subsequent two measurements differed by more than 5 mmHg, additional measurements were performed until two consecutive measurements differed by ≤5 mmHg and an average was recorded.

#### Dietary records

Weighed 7-d diet records were used to evaluate habitual Ca intake with the assumption that it would reflect a long-term Ca intake. The participants’ dietary intakes of energy, macronutrients and micronutrients were calculated as a daily average from the 7-d diet records. Participants registered all foods and supplied information on brand names, cooking and processing. Whenever possible, foods were weighed; otherwise, household measures were applied. The dietary registrations were assessed by the computer database of foods from the National Food Agency of Denmark (Dankost 3000; National Food Agency of Denmark).

#### Faeces

Before analysis, faeces samples were weighed, homogenised and freeze-dried, and all samples from the 5-d collection period were pooled. Faecal energy was measured in duplicate by a bomb calorimeter (IKA Calorimeter System C4000) and the average was recorded. Faecal samples were hydrolysed with 3 m-HCl and thereafter fat excretion was measured by the acidic Bligh & Dyer extraction^(^^64^^)^. Before analysis of faecal Ca content, samples were dried at 525°C for 6 h, ashes were dissolved in 6·5 % HNO_3_ and the solution was diluted with a lanthanum chloride solution. To determine Ca content, samples were measured by atomic absorption spectrophotometry using a PYE UNICAM SP9 atomic absorption spectrophotometer (Philips Electron Optics).

#### Urine

During urine collection, participants ingested tablets of 80 mg *para*-aminobenzoic acid three times per d (240 mg/d). Before analysis, weight and density of the collected urine were measured to calculate the volume. Excretion of urinary Ca was measured using photometric analysis (ABX Pentra 400 analyser; HORIBA ABX S.A.S.) and urinary N using the modified Dumas method with a VarioMax CN analyser (Elementar).

#### Blood samples

Fasting blood samples for the analysis of serum lipids (total CHOL, LDL-CHOL, HDL-CHOL and TAG) were collected in serum tubes and kept at room temperature for 30 min to coagulate. Blood samples for plasma analysis of NEFA were collected in EDTA-containing tubes and immediately placed on ice. All samples were centrifuged at 2500 ***g*** for 10 min at 4°C and stored at −80°C. Total CHOL and TAG were measured by enzymic photometric tests, and HDL-CHOL, LDL-CHOL and NEFA by enzymic colorimetric tests using a Pentra 400 Analyser (HORIBA ABX). For total CHOL, intra-assay CV was 1·0 %, whereas inter-assay CV was 1·7 %. For HDL-CHOL, intra-assay CV was 1·2 %, whereas inter-assay CV was 5·6 %. For LDL-CHOL, intra-assay CV was 2·4 %, whereas inter-assay CV was 3·7 %. For NEFA, intra-assay CV was 1·7 %, whereas inter-assay CV was 5·1 %. For TAG, intra-assay CV was 3·0 %, whereas inter-assay CV was 3·8 %.

### Statistical analyses

Before initiation of trial I, a power calculation was performed using data from other studies. The mean for Ca intake in Denmark was assumed to be 967 (sd 394) mg/d^(^^65^^)^ and the standard deviation for the faecal fat excretion was assumed to be 2 g/d^(^^53^^)^. Based on the standard deviation for fat excretion a study with 160 included participants would have a power of 85 % (accounting for a dropout rate of 10 %) to detect an association between Ca intake and faecal fat excretion if the faecal fat excretion increased 1 g/d per 1000 mg/d increase in Ca intake. Later, a meta-analysis^(^^39^^)^ found that an increase of 800–6000 mg Ca per d increased the faecal fat excretion by a standardised mean difference of 0·99 (95 % CI 0·63, 1·34), corresponding to about 2 g/d.

All statistical analyses were performed using STATA (version 10.1). The level of significance was set at *P* < 0·05.

The collected data were examined for means, ranges and outliers. Normally distributed data are presented as means and standard deviations, and non-normally distributed data by medians, and first and third quartiles (Q1 and Q3). Categorical data are presented by the number of participants in the group. Associations between two parameters (measurements of Ca, fat and energy) were examined by linear regression. Adjusted *R*^2^ was used to assess how well the linear regression model predicted the dependent variable. All models were visually inspected by plot of residuals and occasionally the dependent variable was transformed to obtain normality of residuals. Cook's distance was used to identify observations that deviated from the main population; however, none of the observations had a Cook's distance value ≥1.

To analyse the association between dietary Ca intake (g/d) and the dependent variable faecal fat excretion (g/d) a general multiple linear regression (model I) was built containing the covariates of BMI, age, sex and intake of Ca-containing supplements (yes/no). Afterwards, this model was further adjusted for energy and fat intakes; thus Ca intake was given as g/10 MJ per d and faecal fat excretion as a percentage of the fat intake (model II). Further, adjustments for fibre intake and the interaction fibre × Ca (model III) were tested because increased fibre intake^(^^66^^)^ and possibly also an interaction between fibre and Ca^(^^67^^)^ have been linked to increased faecal fat excretion.

To analyse the association between dietary Ca intake and the dependent variable faecal energy excretion (or blood lipid concentration (mmol/l or μmol/l) or BP (mmHg)) a general multiple linear regression (model I) with covariates as described above was built. This model was further adjusted for energy intake; thus Ca intake was given as g/10 MJ per d (for investigation of faecal energy excretion this variable was given as a percentage of total energy intake). Further adjustments for fibre and the interaction fibre × Ca (model III) were performed as described above.

The possibility that faecal excretion of fat or energy could mediate Ca's association with blood lipid profile was tested by a mediation analysis described by MacKinnon *et al.*^(^^68^^)^.

## Results

**Fig. 1.**
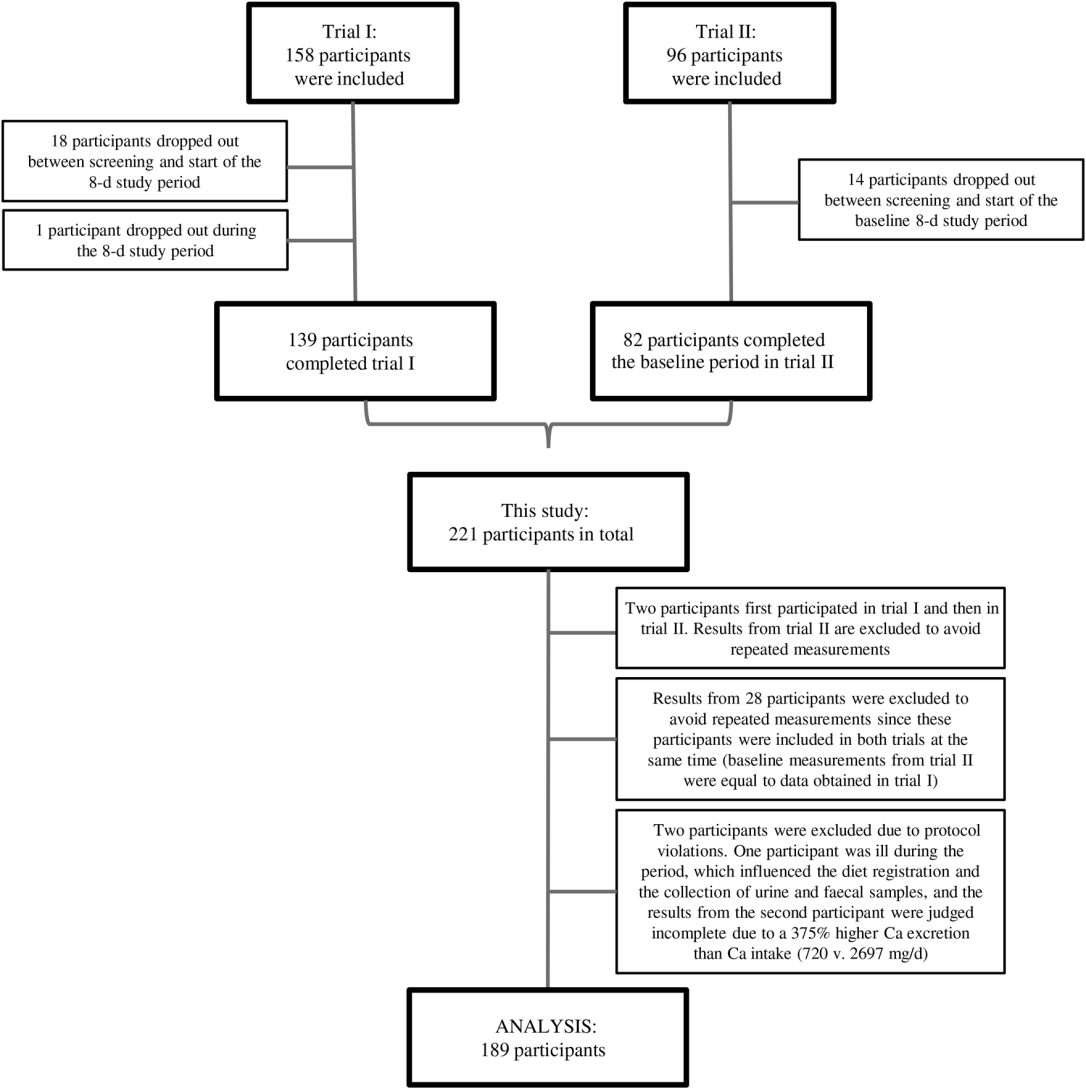
Flow chart. A total of 158 and ninety-six participants were recruited to trials I and II, respectively. In all, eighteen participants in trial I and fourteen participants in trial II dropped out before the 8-d data collection period started and one participant in trial I dropped out during the data collection period. Hence, 221 participants completed the studies. Before data analysis, thirty participants were excluded to avoid repeated measurement from those who participated in both studies and two participants were excluded due to protocol violation.

In total, 189 participants (129 women and sixty men; Table 1) were included in the final data analysis (Fig. 1). Description of faecal and urine samples is found in Table 2.

**Table 1.** Description of the study population (*n* 189) (Medians with quartile 1 (Q1) and quartile 3 (Q3); mean values and standard deviations, unless otherwise specified)

Characteristics	Median	Q1, Q3
Age (years)	31	24, 44
Anthropometry		
Height (m)*		
Mean	1·72
sd	0·1
Weight (kg)*	76·4	68·5, 88·9
BMI (kg/m^2^)*	26·3	22·3, 31·0
Blood pressure		
Systolic blood pressure (mmHg)*	118	111, 125
Diastolic blood pressure (mmHg)*		
Mean	76·5
sd	10·2
Pulse (beats/min)^†^	60	55, 65
Lipid profile		
Total cholesterol (mmol/l)^‡^		
Mean	4·80
sd	1·04
HDL cholesterol (mmol/l)^‡^	1·38	1·14, 1·60
LDL cholesterol (mmol/l)*	2·66	2·13, 3·29
TAG (mmol/l)^‡^	0·92	0·65, 1·33
NEFA (μmol/l)*	587	439, 735
Diet		
Energy intake (kJ/d)	9744	8392, 11 451
Protein (E%)	15·5	13·9, 17·5
Carbohydrate (E%)		
Mean	48·5
sd	5·22
Fat (E%)		
Mean	32·0
sd	5·49
SFA (g/d)	29·3	22·2, 36·5
MUFA (g/d)	26·2	21·1, 32·8
PUFA (g/d)	13·0	10·6, 16·3
Dietary cholesterol (mg/d)^‡^	308	240, 375
Dietary fibre (g/d)	25·4	20·2, 32·5
Dietary Ca intake (mg/d)^§^	1065	856, 1330
FFQ: Ca ≤800 mg/d (%)^∥^^¶^	50·8

E%, energy percentage.

* *n* 187.

† *n* 161.

‡ *n* 188.

§ Habitual Ca intake assessed by a 7-d diet record (without supplements).

∥ Value given as a percentage.

¶ Habitual Ca intake assessed by FFQ.

**Table 2.** Description of faecal and urine samples (*n* 189) (Medians with quartile 1 (Q1) and quartile 3 (Q3))

	Median	Q1, Q3
Faecal dry weight (g/d)	37·6	30·5, 48·7
Faecal wet weight (g/d)	148	113, 210
Faecal water content (%)	74·0	71·0, 77·0
Faecal energy excretion (kJ/d)*	800	630, 1044
Faecal fat excretion (g/d)*	7·68	5·65, 10·0
Faecal Ca excretion (mg/d)*	849	637, 1126
Urinary Ca excretion (mg/d)*	134	90·0, 203
Total Ca excretion (mg/d)^†^	1044	760, 1286

* *n* 186.

† *n* 183.

The median faecal and urinary Ca excretion (Table 2) corresponded to a 98 (sd 31) % excretion of the estimated dietary Ca intake. There was a positive association between dietary Ca intake and total Ca excretion (*β* 0·72 (95 % CI 0·59, 0·86), *P* < 0·001, *R*^2^ 0·39), and positive associations between faecal excretion of Ca and fat (*β* 0·32 (95 % CI 0·26, 0·38), *P* < 0·001, *R*^2^ 0·37) and between faecal excretion of Ca and energy (*β* 10·8 (95 % CI 9·19, 12·5), *P* < 0·001, *R*^2^ 0·48) (Supplementary Fig. S1).

Dietary Ca intake (g/d) was associated with faecal fat excretion (*β* 0·20 (95 % CI 0·12, 0·29); *P* < 0·001). For each 1000 mg/d increase in Ca intake, faecal fat excretion was predicted to increase by 59 (95 % CI 31, 94) %, which means that faecal fat excretion may increase from, for example, 5·7 g/d (Q1) to 9·0 g/d. After adjustment for fat and energy intakes a 1000 mg/10 MJ per d increase in Ca intake was predicted to yield a 31 % higher fat percentage excretion (*P* = 0·004) (Table 3). For example, a 6·6 % excretion of the total dietary fat intake (Q1) would increase to 8·7 %.

**Table 3.** Determinants of faecal fat excretion* (*β* Values and 95 % confidence intervals)

	Model II^†^ (*n* 182)			Model III^‡^ (*n* 182)		
Log (fat % (%))	*β*	95 % CI	*P*	*β*	95 % CI	*P*
Ca intake (Δ1 g/10 MJ per d)	0·12	0·04, 0·20	0·004	0·05	−0·02, 0·12	0·19
Age (Δ10 years)	0·01	−0·02, 0·04	0·67	−0·01	−0·03, 0·02	0·67
Sex						
Women	Ref.			Ref.		
Men	−0·02	−0·08, 0·05	0·64	0·01	−0·04, 0·07	0·63
BMI (Δ5 kg/m^2^)	−0·03	−0·06, 0·01	0·10	−0·01	−0·04, 0·03	0·74
Ca supplements						
No	Ref.			Ref.		
Yes	0·01	−0·07, 0·08	0·85	0·01	−0·06, 0·07	0·85
Dietary fibre (Δ5 g/10 MJ per d)	–	–	–	0·06	0·04, 0·07	<0·001

Fat %, percentage fat excreted; Ref., reference value.

* All *β* values and 95 % confidence intervals in the table are raw data, i.e. data have not been back-transformed.

† Percentage fat excreted (fat %) (calculated as faecal fat excreted/fat intake *×* 100) was log-transformed and analysed by linear regression model adjusted for age, sex, BMI and supplements containing Ca to investigate the effect of Ca intake (g/10 MJ per d).

‡ Model II + additional adjustment for fibre intake. The interaction fibre × Ca was not significant (*P* = 0·95) and therefore removed from the model.

Dietary Ca intake (g/d) was associated with energy excretion (*β* 5·74 (95 % CI 3·26, 8·22); *P* < 0·001). For a woman with a dietary Ca intake of 856 mg/d (Q1) (median age and BMI, without consumption of Ca supplements), a 1000 mg/d increase in Ca intake was related to an increase in faecal energy excretion of 344 (95 % CI 174, 548) kJ/d. After adjustment for energy intake, the predicted effect on faecal energy excretion was reduced (*P* = 0·031) (Table 4). As exemplified above, a 1000 mg/10 MJ per d increase in Ca intake would yield a predicted increase in the energy excretion from 8·3 to 9·7 %, which corresponds to a 133 kJ/d increase for a woman consuming an average energy intake of 9264 kJ/d.

**Table 4.** Determinants of faecal energy excretion* (*β* Values and 95 % confidence intervals)

	Model II^†^ (*n* 182)			Model III^‡^ (*n* 181)		
Square root (energy % (%))	*β*	95 % CI	*P*	*β*	95 % CI	*P*
Ca intake (Δ1 g/10 MJ per d)	0·24	0·02, 0·46	0·031	0·07	−0·14, 0·28	0·50
Age (Δ10 years)	0·05	−0·04, 0·13	0·26	0·02	−0·06, 0·10	0·68
Sex						
Women	Ref.			Ref.		
Men	−0·06	−0·23, 0·12	0·52	0·02	−0·14, 0·18	0·83
BMI (Δ5 kg/m^2^)	−0·09	−0·18, 0·01	0·08	−0·03	−0·12, 0·06	0·57
Ca supplements						
No	Ref.			Ref.		
Yes	0·02	−0·18, 0·22	0·84	0·02	−0·17, 0·20	0·84
Dietary fibre (Δ5 g/10 MJ per d)	–	–	–	0·14	0·10, 0·19	<0·001

Energy %, percentage energy excreted; Ref., reference value.

* All *β* values and 95 % confidence intervals in the table are raw data, i.e. data have not been back-transformed.

† Percentage energy excreted (energy %) (calculated as faecal energy excreted/total energy intake *×* 100) was square root-transformed and analysed by linear regression model adjusted for age, sex, BMI and supplements containing Ca to investigate the effect of Ca intake (g/10 MJ per d).

‡ Model II + additional adjustment for fibre intake. The interaction fibre × Ca was not significant (*P* = 0·99) and therefore removed from the model.

Further adjustments for fibre intake showed no fibre × Ca interaction, either on faecal fat or energy excretion, but adjusting for fibre intake, which was significantly associated with increased faecal fat and faecal energy excretion, abolished the positive association between Ca intake and excretion of faecal fat and energy (Tables 3 and 4). Intake of Ca-containing supplements was not associated with the outcomes in any of the models.

For the lipid profile, we observed that a 1000 mg/d increase in Ca intake (g/d) decreased concentrations of total CHOL by 0·47 (95 % CI 0·11, 0·82) mmol/l (*P* = 0·011), LDL-CHOL by 0·47 (95 % CI 0·17, 0·77) mmol/l (*P* = 0·002) and TAG by 17 (95 % CI 1·6, 30) % (*P* = 0·032), and reduced the total CHOL:HDL-CHOL ratio by 10 (95 % CI 1·4, 18) % (*P* = 0·025) and LDL-CHOL:HDL-CHOL ratio by 16 (95 % CI 2·9, 27) % (*P* = 0·018). After adjustment for energy intake, total CHOL and LDL-CHOL concentrations decreased by increasing Ca intake (Table 5). Adjustment for fibre intake had no significant influence on lipid profile (Table 5). Concentrations of HDL-CHOL, TAG and NEFA, and the total CHOL:HDL-CHOL and LDL-CHOL:HDL-CHOL ratios were not influenced by Ca intake when adjusted for energy intake or by further adjustment for fibre intake (Supplementary Table S1). Mediation analysis showed that faecal excretion of fat or energy did not mediate the effect of Ca intake on lipid profile (Supplementary Table S2).

**Table 5.** Determinants of lipid profile (*β* Values and 95 % confidence intervals)

	Model II*			Model II*			
Total CHOL (mmol/l) (*n* 184)	*β*	95 % CI	*P*	LDL-CHOL (mmol/l) (*n* 183)	*β*	95 % CI	*P*
Ca intake (Δ1 g/10 MJ per d)	−0·62	−0·96,−0·28	<0·001	Ca intake (Δ1 g/10 MJ per d)	−0·49	−0·78, −0·20	0·001
Age (Δ10 years)	0·36	0·22, 0·49	<0·001	Age (Δ10 years)	0·33	0·22, 0·45	<0·001
Sex				Sex			
Women	Ref.			Women	Ref.		
Men	−0·31	−0·58, −0·04	0·027	Men	0·05	−0·19, 0·28	0·70
BMI (Δ5 kg/m^2^)	0·20	0·05, 0·34	0·011	BMI (Δ5 kg/m^2^)	0·19	0·06, 0·31	0·004
Ca supplements				Ca supplements			
No	Ref.			No	Ref.		
Yes	0·05	−0·27, 0·37	0·76	Yes	0·02	−0·25, 0·29	0·89

CHOL, cholesterol; Ref., reference value.

* Lipid outcome analysed by linear regression model adjusted for age, sex, BMI, supplements containing Ca and energy intake to investigate the effect of Ca intake (g/10 MJ per d). Additional adjusting for interaction fibre × Ca was not significant (total CHOL *P* = 0·48, LDL-CHOL: *P* = 0·28) and therefore removed from the model. Neither had fibre intake a significant influence on the models (total CHOL *P* = 0·14, LDL-CHOL *P* = 0·21) and model III for total CHOL and LDL-CHOL were therefore not different from model II.

Ca intake did not influence systolic BP (*P* = 0·11) or diastolic BP (*P* = 0·25). However, when the models were energy adjusted, systolic BP decreased 5·2 (95 % CI 1·8, 8·4) % per 1000 mg/10 MJ per d increase in Ca intake (*P* = 0·003).

## Discussion

We observed that a higher Ca intake was associated with a more beneficial lipid profile, i.e. decreased concentrations of total CHOL and LDL-CHOL as well as a lower systolic BP. Although a higher Ca intake was associated with higher faecal fat and energy excretion in the models adjusted for energy, BMI, age, sex and intake of Ca-containing supplements, the associations disappeared when adjusted for dietary fibre intake. Thus, the association with blood lipids did not seem to be mediated by higher faecal fat or energy excretion.

A meta-analysis of fifteen RCT found that a daily 800–6000 mg higher Ca intake increased the faecal fat excretion by 2 g/d, corresponding to a 37 % increase^(^^39^^)^. There was no difference in the effect between studies using dairy products *v*. Ca supplements; however, the heterogeneity of the overall analysis (*I*^2^ = 49·5 %) suggests possible confounding factors. In our analysis, a daily 1000 mg/10 MJ higher Ca intake was associated with a 31 % increase in fat excretion. However, this association disappeared when the models were adjusted for fibre intake (a possible confounder) and it is questionable how well the effect of fibre intake is considered in the RCT included in the meta-analysis.

Intervention studies have found that higher Ca intakes from milk or cheese increase faecal fat excretion from 3·9 to 5·2–5·7 g/d corresponding to 33–46 % increase per 800 mg/d Ca^(^^58^^)^, from 5·9 to 8·3 g/d corresponding to a 39 % increase per 1500 mg/d Ca^(^^61^^)^ or tend to increase the faecal fat content from 20 to 23 %^(^^56^^)^. The effects in these studies are higher than in our study but one of the main differences between these studies and our study is the amount of Ca. These studies provided very low daily Ca intake in the control groups (362 mg/10 MJ, 417 mg, 504 mg/10 MJ), compared with our study where the lowest Ca intake was 498 mg/d (557 mg/10 MJ per d), and only 10 % of our participants had a Ca intake below 661 mg/d (774 mg/10 MJ per d). Another observation is the low content of dietary fibres in the intervention diets (18·4–20·3 g/d^(^^58^^)^ or 13·5–15·3 g/10 MJ^(^^61^^)^). In our study the fibre intake was much higher (only 25 % had a fibre intake <20·2 g and only 10 % an intake <17·1 g/10 MJ) and our analysis showed that a 10 g/10 MJ per d increase in fibre intake would increase the faecal fat excretion by 30 %. It can be speculated, as indicated by other authors^(^^39^^,^^49^^)^, that a plateau is reached for the effect of Ca when the Ca intake is as high as in our study population. Another interesting observation is the very different amounts of fat excretion in the RCT^(^^39^^,^^58^^,^^61^^)^. For example, the intake of a milk-based diet (Ca: 1143 mg/d) in one study resulted in a fat excretion of 5·2 g/d^(^^58^^)^, which was very similar to the fat excretion (5·87 g/d) in the control group (Ca: 474–504 mg/10 MJ) of another study^(^^61^^)^. A difference like this is probably related to methods for the determination of faecal fat content or other dietary factors that differed between the studies. From our study it is not clear if the increased fat excretion found in RCT is transient or if we do not observe the association in our free-living population because of the habitual intake of Ca (and fibre) is higher than that provided in intervention diets.

Several studies have shown that increased Ca intake increases faecal energy excretion^(^^58^^–^^61^^)^. The increase in energy excretion observed in other studies ranges from 96 to 203 kJ/d, corresponding to a 13–31 % increase by a 800–1600 mg/d increase in Ca intake^(^^58^^,^^59^^,^^61^^)^, with one study^(^^60^^)^ finding a 361 kJ/d increase (about 53 % increase) by a 1300 mg/d Ca increase. The former studies are similar to our results showing an increase of 133 kJ/d per 1000 mg Ca/10 MJ. However, when adjusting for fibre intake the significant association of Ca disappeared in our study.

Similar to our results, Jacqmain *et al.*^(^^7^^)^ investigated data from 470 persons in the Québec Family Study and found that a higher Ca intake was correlated with a reduced ratio of total CHOL:HDL-CHOL. The ratio of total CHOL:HDL-CHOL for women was significantly reduced by 11·3 % (from 4·16 to 3·69) when the <600 mg/d Ca group was compared with the >1000 mg/d Ca group. In our study, a 1000 mg/d increase in Ca was associated with a 10 % lowered total CHOL:HDL-CHOL ratio; however, this relationship was not significant when adjusted for energy intake. Furthermore, a higher Ca intake was correlated with reduced concentrations of LDL-CHOL and total CHOL^(^^7^^)^ as we also observed. In agreement with the observational results, Reid^(^^69^^)^ reviewed the effect of a higher Ca intake on lipid profile from intervention studies and concluded that higher Ca intake reduced total CHOL and LDL-CHOL concentrations. Furthermore, a few studies indicated an increase in HDL-CHOL concentration^(^^69^^)^ which we did not find. Several interventions^(^^55^^–^^57^^)^ have found that a cheese diet (+800 mg/d Ca) reduced concentrations of total CHOL by 0·21–0·26 mmol/l and LDL-CHOL by 0·20–0·22 mmol/l, compared with a butter diet. In the present study, we found an association of a 0·62 mmol/l reduction in total CHOL concentration per 1000 mg/10 MJ per d increase in Ca intake, which is a larger predicted effect than that observed in other studies^(^^55^^–^^57^^)^. Furthermore, LDL-CHOL concentration was associated with a 0·49 mmol/l reduction per 1000 mg/10 MJ per d increase in Ca intake and again a larger predicted effect than observed in the intervention studies^(^^55^^–^^57^^)^. In our study, increased Ca intake did not increase HDL-CHOL concentration, and the reduced TAG concentration as well as the reduced ratios of total CHOL:HDL-CHOL and LDL-CHOL:HDL-CHOL disappeared when we adjusted for energy intake. These latter observations add to the inconsistency in these lipid markers where only a few studies^(^^50^^,^^56^^)^ have found effects of increasing Ca intake. In contrast, Zemel *et al.*^(^^15^^)^ and Jacobsen *et al*.^(^^60^^)^ did not find either long-term or short-term periods on low *v*. high dairy consumption to affect lipid profile and Boon *et al.*^(^^50^^)^ only found an effect on TAG concentration.

Many interventions are not directly comparable with our study since they provided diets with a high fat^(^^54^^)^ or high SFA^(^^48^^,^^49^^,^^58^^)^ content; thus the CHOL concentrations were expected to increase during the intervention despite the higher Ca intake. Increased Ca intake (800–1500 mg/d) reduced the total CHOL and LDL-CHOL concentrations measured after the intervention in the high Ca group, compared with the low Ca groups^(^^54^^,^^58^^)^. Comparable with our results, concentrations of total CHOL differed by 0·29–0·48 mmol/l and LDL-CHOL by 0·33–0·46 mmol/l^(^^54^^,^^58^^)^. These effects have also been observed by Ca-fortified products where a 1011 mg/d increase in Ca intake decreased total CHOL concentration by 0·24 mmol/l and tended to decrease LDL-CHOL concentration by 0·14 mmol/l^(^^49^^)^. Furthermore, a 900 mg/d increase in Ca resulted in a 0·33 mmol/l reduction in LDL-CHOL concentration^(^^48^^)^. The majority of studies investigating lipid profile are in agreement with our results showing that an increased Ca intake reduces total CHOL and LDL-CHOL concentrations. However, some studies comparing more than one intervention diet did not always find a clear dose–response pattern in the Ca effect within their study^(^^50^^,^^55^^,^^57^^,^^60^^)^ and we did not observe a clear dose–response pattern when we grouped participants into tertiles based on their Ca intake (data not shown). This could indicate that there are unexplained effects related to the food matrix.

We observed an association of a 5·2 % reduction in systolic BP per 1000 mg/10 MJ per d increase in Ca intake with no relation to diastolic BP. This prediction is higher than findings from two meta-analyses^(^^70^^,^^71^^)^ that both found a smaller (0·9–1·4 mmHg) effect on systolic BP.

In general, the predicted effects of Ca intake on blood lipid profile found in our free-living population are in agreement with effects found in intervention studies; however, it is important to consider the effect size. Elevated LDL-CHOL concentration is associated with an increased risk of CVD and a reduction of 1·0 mmol/l is estimated to reduce all-cause mortality by 10 % and death related to CVD by 20 %^(^^41^^)^. This reduction of LDL-CHOL concentration by entirely increasing Ca intake will in our study require a 2 g/d increase in Ca intake, which does not seem realistic when the habitual Ca intake for many Danes is relatively high. However, increasing Ca intake may be considered as a part of a preventive healthy diet for those with a habitual low Ca intake.

The major limitation in this study is that the study population does not necessarily reflect the general Danish population. In Denmark, habitual Ca intake is high and to obtain associations over a large range in Ca intake, we aimed at recruiting 50 % of the participants with a Ca intake below 800 mg/d. To obtain data from participants with a low Ca intake, we included baseline data from participants in trial II that were collected in the same time period and by the exact same methods as in trial I. The inclusion of data from fifty extra participants broadens the Ca range, thus reducing the possibility of making a type II error. Furthermore, we did not find an association of Ca-containing supplements, which could be caused by the fact that (1) too few of our participants consumed supplements, (2) the effect of additional Ca from supplements reached a Ca threshold or (3) the information obtained about intake of Ca supplementation was too limited. In total, thirty-three participants consumed Ca supplements in the present study and excluding these participants from the analyses did not alter the results.

The major strength of this study is that faeces and urine samples were collected for 5 and 2 d, respectively. The median (Q1, Q3) percentage of relative Ca excretion (urine and faecal Ca excretion/Ca intake) was 94 (79, 115) %, indicating good concordance between Ca measurements from the urine and faecal samples and the diet registration. Additionally, we included data that allowed us to investigate the association of dietary fibre, which may be a potential confounder of the effect of Ca intake in relation to energy and fat excretion in our population.

In conclusion, dietary Ca intake is negatively associated with total CHOL and LDL-CHOL concentrations and systolic BP. Under free-living conditions, these associations do not seem to be mediated by increased faecal excretion of fat or energy.

## Supplementary material

For supplementary material accompanying this paper visit http://dx.doi.org/10.1017/jns.2017.55. click here to view supplementary material
