# Supplementary material for: Calcium intake and the associations with faecal fat and energy excretion, and lipid profile in a free-living population
Source: J Nutr Sci. 2017 Sep 19;6:e50. doi: 10.1017/jns.2017.55 (PMC5672323; doi:10.1017/jns.2017.55)

Supplementary Figure 1: Associations between A) dietary calcium intake and total calcium excretion (urine + faeces), B) faecal calcium excretion and faecal fat excretion, and C) faecal calcium excretion and faecal energy excretion.

A)

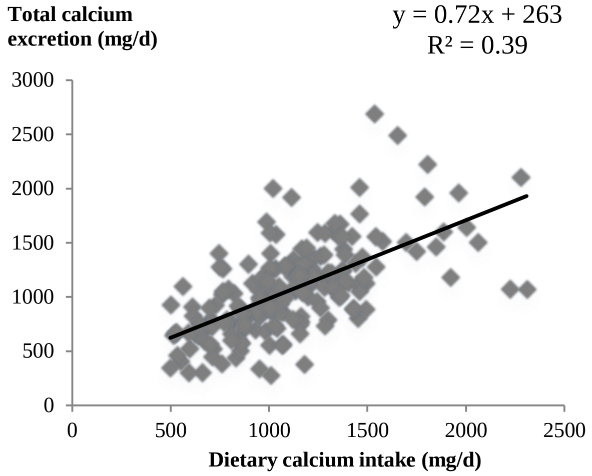

B)

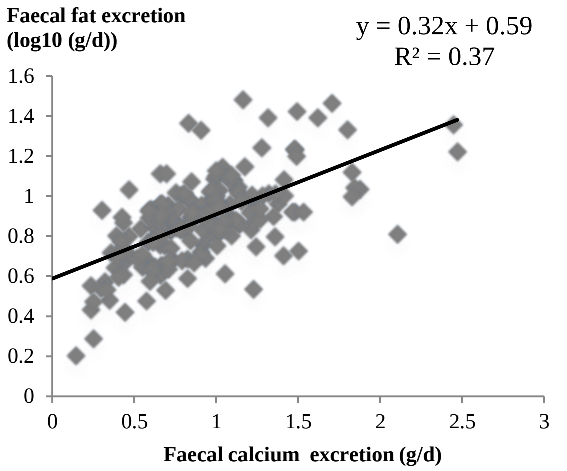

C)

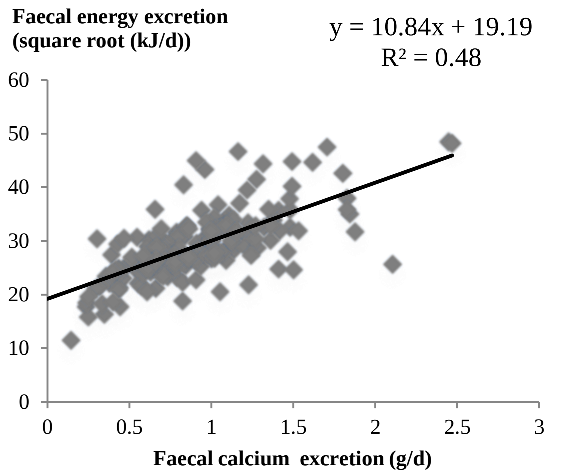

Supplement: Supplementary file 1 [file S2048679017000556sup.zip › S2048679017000556sup001.pdf]
