# Supplementary material for: Calcium intake and the associations with faecal fat and energy excretion, and lipid profile in a free-living population
Source: J Nutr Sci. 2017 Sep 19;6:e50. doi: 10.1017/jns.2017.55 (PMC5672323; doi:10.1017/jns.2017.55)
Supplement: Supplementary file 1 [file S2048679017000556sup.zip › S2048679017000556sup002.doc]

**Supplementary Table S1.** Determinants of lipid profile

(β-Values, 95% confidence intervals and *P*-values. All β-values and 95% confidence intervals in the table are raw data, i.e. data has not been back-transformed)

|  |  | Model II ‡ |  |
| --- | --- | --- | --- |
|  | β | 95% CI | *P* |
| **Log 10 (HDL-CHOL)** * |  | (n=184) |  |
| **Calcium intake (Δ 1g/10MJ/d)** | **-0.03** | **-0.06 ; 0.01** | **0.19** |
| Age (Δ10 years) | 0.00 | -0.01 ; 0.02 | 0.98 |
| Sex |  |  |  |
| Women |  | Ref. |  |
| Men | -0.11 | -0.14 ; -0.08 | <0.001 |
| BMI (Δ5 kg/m^2^) | -0.03 | -0.04 ;- 0.01 | 0.002 |
| Calcium supplements |  |  |  |
| No |  | Ref. |  |
| Yes | 0.00 | -0.03 ; 0.04 | 0.81 |
| **Log 10 (TAG)** * |  | (n=184) |  |
| **Calcium intake (Δ 1g/10MJ/d)** | **-0.05** | **-0.12 ; 0.03** | **0.21** |
| Age (Δ10 years) | -0.00 | -0.03 ; 0.03 | 0.88 |
| Sex |  |  |  |
| Women |  | Ref. |  |
| Men | 0.01 | -0.05 ; 0.07 | 0.74 |
| BMI (Δ5 kg/m^2^) | 0.09 | 0.06 ; 0.12 | <0.001 |
| Calcium supplements |  |  |  |
| No |  | Ref. |  |
| Yes | -0.01 | -0.08 ; 0.06 | 0.73 |
| **Square root (NEFA)** † |  | (n=183) |  |
| **Calcium intake (Δ 1g/10MJ/d)** | **0.14** | **-1.79 ; 2.06** | **0.89** |
| Age (Δ10 years) | 0.92 | 0.16 ; 1.68 | 0.018 |
| Sex |  |  |  |
| Women |  | Ref. |  |
| Men | -0.46 | -2.00 ; 1.09 | 0.56 |
| BMI (Δ5 kg/m^2^) | 0.86 | 0.01 ; 1.70 | 0.047 |
| Calcium supplements |  |  |  |
| No |  | Ref. |  |
| Yes | -1.20 | -2.99 ; 0.60 | 0.19 |
| **Log10 (Total/HDL-CHOL)** * |  | (n=184) |  |
| **Calcium intake (Δ 1g/10MJ/d)** | **-0.03** | **-0.07 ; 0.01** | **0.16** |
| Age (Δ10 years) | 0.03 | 0.01 ; 0.04 | <0.001 |
| Sex |  |  |  |
| Women |  | Ref. |  |
| Men | 0.08 | 0.05 ; 0.11 | <0.001 |
| BMI (Δ5 kg/m^2^) | 0.05 | 0.03 ; 0.06 | <0.001 |
| Calcium supplements |  |  |  |
| No |  | Ref. |  |
| Yes | 0.00 | -0.04 ; 0.04 | 0.97 |
| **Log10 (LDL/HDL-CHOL)** * |  | (n=184) |  |
| **Calcium intake (Δ 1g/10MJ/d)** | **-0.05** | **-0.11 ; 0.01** | **0.12** |
| Age (Δ10 years) | 0.05 | 0.02 ; 0.07 | <0.001 |
| Sex |  |  |  |
| Women |  | Ref. |  |
| Men | 0.11 | 0.06 ; 0.16 | <0.001 |
| BMI (Δ5 kg/m^2^) | 0.06 | 0.03 ; 0.08 | <0.001 |
| Calcium supplements |  |  |  |
| No |  | Ref. |  |
| Yes | -0.00 | -0.06 ; 0.06 | 0.99 |

CHOL, cholesterol; Ref., reference value;
* Variable was log-transformed
† Variable was square root-transformed
‡ Lipid outcome analysed by linear regression model adjusted for age, sex, BMI, supplements containing calcium to investigate the effect of calcium intake (g/10MJ/d). Additional adjusting for interaction fibre×calcium was not significant (HDL-CHOL: p=0.13, TAG: p=0.22, NEFA: p=0.56, Total/HDL-CHOL: p=0.062, LDL/HDL-CHOL: p=0.12) and therefore removed from the model. Neither had fibre intake a significant influence on the models (HDL-CHOL: p=0.92, TAG: p=0.29, NEFA: p=0.69, Total/HDL-CHOL: p=0.29, LDL/HDL-CHOL: p=0.29) and the model III were therefore not different from model II.

**Supplementary Table S2.** Mediation analysis (coefficients and their *P*-values). The regressions are further adjusted for the covariates: age, sex, BMI and intake of calcium supplementation

|  |  | |  |  | | | |  |  | |  |  |
| --- | --- | --- | --- | --- | --- | --- | --- | --- | --- | --- | --- | --- |
|  | ***c*** | ***P*** |  | ***c'*** | ***P*** | ***b*** | ***P*** |  | ***a*** | ***P*** |  |  |
| Y:Total cholesterol (mmol/L) X: Calcium intake (g/10MJ/d) M: Faecal fat excretion (g/d) | -0.62 | <0.001 |  | -0.64 | <0.001 | -0.02 | 0.2 |  | -0.22 | 0.8 |  | -2.8 |
| Y:Total cholesterol (mmol/L) X: Calcium intake (g/10MJ/d) M: Faecal energy excretion (kJ/d/100) | -0.62 | <0.001 |  | -0.64 | <0.001 | -0.02 | 0.2 |  | -0.19 | 0.8 |  | -2.9 |
| Y: LDL cholesterol (mmol/L) X: Calcium intake (g/10MJ/d) M: Faecal fat excretion (g/d) | -0.49 | 0.001 |  | -0.51 | 0.001 | -0.02 | 0.08 |  | -0.22 | 0.8 |  | -3.4 |
| Y: LDL cholesterol (mmol/L) X: Calcium intake (g/10MJ/d) M: Faecal energy excretion (kJ/d/100) | -0.49 | 0.001 |  | -0.51 | 0.001 | -0.02 | 0.09 |  | -0.19 | 0.8 |  | -3.6 |
